# Gender Based Within-Household Inequality in Childhood Immunization in India: Changes over Time and across Regions

**DOI:** 10.1371/journal.pone.0035045

**Published:** 2012-04-11

**Authors:** Ashish Singh

**Affiliations:** Indira Gandhi Institute of Development Research, Mumbai, Maharashtra, India; Louisiana State University Health Sciences Center, United States of America

## Abstract

**Background and Objectives:**

Despite India's substantial economic growth in the past two decades, girls in India are discriminated against in access to preventive healthcare including immunizations. Surprisingly, no study has assessed the contribution of gender based within-household discrimination to the overall inequality in immunization status of Indian children. This study therefore has two objectives: to estimate the gender based within-household inequality (GWHI) in immunization status of Indian children and to examine the inter-regional and inter-temporal variations in the GWHI.

**Data and Methods:**

The present study used households with a pair of male-female siblings (aged 1–5 years) from two rounds of National Family Health Survey (NFHS, 1992–93 and 2005–06). The overall inequality in the immunization status (after controlling for age and birth order) of children was decomposed into within-households and between-households components using Mean log deviation to obtain the GWHI component. The analysis was conducted at the all-India level as well as for six specified geographical regions and at two time points (1992–93 and 2005–06). Household fixed-effects models for immunization status of children were also estimated.

**Results and Conclusions:**

Findings from household fixed effects analysis indicated that the immunization scores of girls were significantly lower than that of boys. The inequality decompositions revealed that, at the all-India level, the absolute level of GWHI in immunization status decreased from 0.035 in 1992–93 to 0.023 in 2005–06. However, as a percentage of total inequality, it increased marginally (15.5% to 16.5%). In absolute terms, GWHI decreased in all the regions except in the North-East. But, as a percentage of total inequality it increased in the North-Eastern, Western and Southern regions. The main conclusions are the following: GWHI contributes substantially to the overall inequality in immunization status of Indian children; and though the overall inequality in immunization status declined in all the regions, the changes in GWHI were mixed.

## Introduction

Pronounced gender bias exists in most of the countries of South Asia [1]–[3]. Extant literature on the subject has identified “preference for sons over daughters” as the reason for the gender bias against girls in South Asian countries, particularly India. As per the same body of literature, this preference for sons over daughters manifests itself in the form of discrimination against daughters in providence for food, health care and education [4]–[24], and ultimately for excess female child mortality rates [4], [5], [25]–[37]. Preference for sons has also been associated with preferential abortion of female fetuses and even to female infanticide [38]–[40].

The past studies have also documented the reasons behind the preference for sons over daughters in the context of Indian subcontinent. They have found that sons are preferred over daughters for a number of economic, social and religious reasons (perceived greater economic, social, and religious utility of sons than of daughters), including financial support, old age security, property inheritance, dowry, family lineage, prestige and power, birth and death rituals, and beliefs about religious duties and salvation [4], [6], [9], [10], [26], [27], [29], [38], [41]–[52]. “Parents of girls are socially bound to find grooms for their daughters and often pay all the marriage expenses (including dowry); social customs and norms dictate that parents cannot expect much support (emotional or economic) from married daughters. In contrast, parents expect sons to provide financial and emotional care and regard them as a social security for old age, inheritance laws largely favor sons and sons perform important religious roles, ensure the continuation of the family lineage, and are desired to increase a family's capacity to defend itself or to exercise power [18 (p.396)], [29], [46], [53]–[61]”.

The gender based discrimination in providence for basic necessities like immunization and nutrition in India, leads to gender based inequality in immunization and nutritional status among Indian children. Though some of the earlier studies have focused upon gender based differentials in nutrition and immunization [8], [15], [18], they have not documented the contribution of gender based discrimination within the households to the overall inequality in immunization or nutrition status among the Indian children. The studies on gender based differentials in nutrition and immunization invariably used logistic regression models and reported (based on the odds ratios) that the male children were more likely to receive full immunization or minimum nutrition than the female children, in the whole population [8], [15], [18]. This is an important reporting but this kind of analysis compares all the female children with all the male children in the sample. In simple terms, it compares a female child of one household not only with the male children in the same household but also with the male children of the other households and vice versa. Using this kind of analysis, one cannot tell what proportion of the gender differential in the observed outcome variable (say, immunization status or nutrition) is due to the direct discrimination between girls and boys within the households. The investigation of gender based within household discrimination is important because it is taking place inside the house and it is almost impossible for the governmental bodies (law enforcement, social reforms and policy making) to either directly identify it or to estimate its extent.

I in the present study, therefore, estimated gender based within-household inequality in the immunization status of Indian children aged 1–5 years using a novel inequality decomposition technique and data from a national level survey. To be specific, I investigated the following two questions: first, what is the extent of gender based within-household inequality (GWHI) in the immunization status of Indian children and second, what is the extent of inter-regional variations as well as the changes over time in the GWHI. Immunization status is chosen because it is an important indicator of preventive health care utilization [15], [62] and its absence can be linked to increased mortality risks and functional impairments in adulthood. Vaccine-preventable diseases are responsible for nearly 20% of the 8.8 million deaths occurring annually among children under five years of age. An estimated 23 million children under the age of one were not vaccinated in 2009; 70% of these children live in ten countries, one of which is India [63]. Immunization status is also an indicator of progress towards the child health targets established under the Millennium Development Goals [64]. Further, reducing child mortality and achieving the millennium development goal for child survival depends on whether effective and sustainable interventions (including immunizations) can be delivered to high proportions of children and mothers [62], [65].

Simple but innovative inequality decomposition technique was used to carry out the decompositions of overall inequality in immunization status of children into within-households and between- households components. The decomposition was carried out at the all-India level and for the six specified geographical regions of India, at two time points (1992–93 and 2005–06). To this effect, national level data on child immunization status from two cross-sectional surveys conducted in 1992–93 (NFHS-1) and 2005–06 (NFHS-3) were used. This helps in understanding the changes in gender based within household inequality across the six regions over a period of thirteen years or so.

## Methods

### Ethics statement

The data were analyzed anonymously, using publicly available secondary data; therefore no ethics review is required for this work.

### Study Settings and Data

The present study had two major objectives: first, to estimate the extent of gender based within-household inequality (GWHI) in immunization status for Indian children and second, to examine the inter-regional variations and the changes over time in the GWHI. For this purpose, such data at two time points were needed which were sufficiently apart (time-wise) and which were sufficiently large to permit analysis at the regional level apart from the all-India level. Also, for a comparison of the estimates over time, the sources of data at the two time points should have comparable sampling designs.

The data for the present study is taken from two cross-sectional rounds of National Family Health Survey (NFHS) conducted during 1992–93 and 2005–06. These surveys are nationally representative and cover more than 99% of the Indian population. The household and eligible female informant response rates were consistently above 90% in both the NFHS rounds. The NFHS followed Stratified Probability Proportional to Size (PPS) systematic sampling design. These surveys are the Indian version of the Demographic Health Survey (DHS), and provide consistent and reliable estimates of fertility, mortality, family planning, utilization of maternal and child health care services and other related indicators at both the national and state levels. The NFHS uses standard model questionnaires designed for, and widely used in, developing countries [66]. Details of these nationally representative surveys have been described in their respective reports [67], [68]. The estimates obtained from the two rounds of NFHS are comparable because both the rounds followed comparable sampling design to select households and individuals for the interview [15], [69]. I used data from the interviews with women of reproductive age which includes information about their children. It is worthwhile to note that all the children covered in the survey were born to the interviewed women and none of them were parentless.

India is comprised of 29 states and seven Union Territories. The different states of India are at different levels of socio-economic development; most of the western and southern states of India are economically and demographically advanced than the northern and eastern states of India [70]–[72]. So, any meaningful analysis should take into account the vast regional diversity present in India. To take care of this regional diversity, present analysis was carried out for India as a whole and separately for the six major geographic regions of India namely North, Central, East, North-east, West, and South. Northern region comprises of states of Jammu & Kashmir, Himachal Pradesh, Delhi, Uttaranchal, Punjab, Haryana and Rajasthan. The states of Uttar Pradesh, Madhya Pradesh and Chattisgarh come under the central region. The Eastern region comprises of states of Bihar, Jharkhand, West Bengal and Orissa. The North-eastern region includes the seven north-eastern sister states namely Assam, Arunachal Pradesh, Meghalaya, Manipur, Tripura, Nagaland and Sikkim. The Western region includes states of Maharashtra, Goa and Gujarat. Finally, the Southern region comprises of states of Andhra Pradesh, Karnataka, Kerala, Tamil Nadu and Pondicherry. This categorization of states into regions follows the categorization provided in the respective NFHS reports as well as earlier studies in similar context [67]–[68], [73].

Since, the interest of the study is in gender based within-household inequalities, the eligible sample comprises of those households which had at least one pair of male-female children under the age of 5 years. The total number of households with at least a male-female pair of children were 1972 (i.e., the eligible sample) and 3930 in 1992–93 and 2005–06 respectively. Of these there were 1934 and 3653 households with exactly one male-female pair of children in 1992–93 and 2005–06 respectively. These households which comprise of 98 percent of the eligible sample in 1992–93 and 93 percent of the eligible sample in 2005–06 were used in the analysis.

It may be noted that, in 2005–06, in the full sample (all children aged 1–5 years), the proportion of male and female children were 52.6 and 47.4%, respectively. In the remaining households (not included in the analysis), the proportions were 53.3 and 46.7%, respectively. Similarly, for 1992–93, the proportions of male and female children in the full sample were 51.1% and 48.9%. In the remaining households, the proportions were 51.3 and 48.7%, respectively. Therefore, in both the years, the sex ratio in the full sample was similar to that of the analyzed sample as well as to that of the excluded households.

### Immunization Status

The outcome of interest in the present study is immunization status of children aged 12 months to 4 years in 1992–93 and 12 months to 5 years in 2005–06. The analyses were limited to children aged less than 4 years in 1992–93 and less than 5 years in 2005–06, because of the fact that the data on immunization was only collected for children born in the 4 years and 5 years preceding the 1992–93 and 2005–06 survey rounds, respectively. This difference in the sample is not likely to bias the comparison of the estimates from the two survey rounds because the estimates are obtained after adjusting the immunization status of children for age. The sample was restricted to children above one year because a child requires at least nine months to receive immunizations for the six vaccine-preventable diseases (namely, tuberculosis, diphtheria, whooping cough, tetanus, polio, and measles). BCG (for tuberculosis) should be given at birth or at first clinical contact, DPT (for diphtheria, whooping cough and tetanus) and Polio require three vaccinations at approximately 4, 8 and 12 weeks of age, and measles should be given at or soon after reaching 9 months of age [68].

In practice and to maximize the benefits of immunization, assessment of completion of immunization for children is generally done between 12 and 24 months after birth. However, the Pulse Polio Immunization Program of Government of India (polio is a major cause of concern in India), which was launched in 1995 [74], focuses on all children aged up to five years and therefore the government uses mass as well as print media extensively for campaigning to pursue the parents to take all of their children aged up to five years to polio immunization administration centers for administration of polio drops. In addition, on designated days for polio drops administration (other than the regular availability at the health centers), the volunteers go door to door for administering polio drops to all the children up to five years of age. I have therefore, included children up to five years of age in the analysis. However, there is a possibility that including children up to five years of age might exaggerate the immunization coverage because mortality due to the (above listed) vaccine preventable diseases might exclusively eliminate the non-vaccinated children from the sample.

The immunization status is computed based on information whether a child has received immunizations of BCG, DPT, Measles and Polio. Each one of them has been given a score of 0 or 1 based on the following: for BCG and Measles, only one dosage each is required, so if a child has received the dosage for BCG, the score assigned for BCG is 1; similarly if a child has received the dosage for Measles, the score assigned for Measles is also 1. For DPT and Polio, three dosages each are required, so if a child has received all the three dosages of DPT, then s/he is considered to have received BCG immunization and therefore a score of 1 or 0 otherwise; similarly for Polio, if a child has received all the three dosages, then s/he is considered to have received immunization against Polio and it is scored as 1 (0 otherwise). The immunization status is the sum of these scores and varies from 0 to 4. A child will have immunization status as 0 when s/he has received incomplete (or no) dosage of DPT and Polio as well as no dosages of BCG and Measles. S/he will have an immunization status of 4 if s/he has received 1 dosage each of BCG and Measles and 3 dosages each of DPT and Polio. In case where the immunization status has a value 4, the child is said to have received the complete recommended set of immunizations.

After computing the immunization status for each child in the sample, I employed two approaches to estimate the extent of gender based within-household inequality in immunization status of the children. The details of these approaches are presented below.

### Household Fixed Effects

To begin with, I used a multiple linear regression model with household fixed effects for each of the survey years to investigate whether girls were discriminated against boys within households when it comes to providing vaccination against six vaccine preventable diseases. The immunization status (*IS*) of a child depends upon his/her personal characteristics (such as gender, birth order and age) and the characteristics of the household where s/he resides (for example, parental education). Some of these household characteristics might be observed while the others may not. Use of household fixed effects makes it possible to control for all unobserved and observed household-level variables which are common to the children (for example, parental education) within a household. Formally the model can be written as:
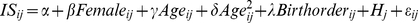
(1)where, *i* stands for the male ( = 0) or female ( = 1) child within the household and *j* stands for the household. “*Female*” stands for the dummy for the sex (male as reference) of the child; “*Age*” and “*Birth order*” for age and birth order of the child respectively; and “*H*” stands for household fixed effects. In this analysis all the household-level variables that are invariant across children (*H*) within a household will automatically drop out. Household fixed-effects have also been used (in different contexts) in past studies [75]–[76].

### Inequality Decomposition

At the second stage, the study used a simple but innovative technique whose basic intuition lies in the fact that the difference between the immunization status of male and female siblings (within a household) may be due to gender, birth order or age [8], [15], [18], [73]. This is so, because all the other factors like parental education or religion are same for both the children within a household. Once the immunization status is corrected for birth order and age, then the sole difference in the immunization status of the children within a household can be attributed to their gender. If the overall inequality in the corrected immunization status is now decomposed into within-households and between-households components, the within-household component can be attributed to gender based within-household inequality in immunization status. A ratio of within-household inequality to the overall inequality will provide the gender based within-household inequality as a fraction of total inequality.

To correct (control for) the immunization status of children for age and birth order, I regressed the actual observed immunization status on age (and age squared) and birth order of the children and used the residuals from this regression. This adjustment (at the all India level) was done separately for the two survey rounds. The corrected immunization status thus represents the immunization status of a child of an “average” age and an “average” birth order. The corrected immunization status is then used in the inequality decomposition exercise. The underlying procedure for carrying out the decomposition is as follows:

The decomposition of overall inequality into within-households (intra-household) and between households (inter-household) is carried out separately for the two survey rounds. For each of the survey round, the analysis is performed separately for India as a whole and for the six geographical regions. For ease of explanation, consider the all India sample of 2005–06. The total sample is partitioned into groups based on households. That is, each household is considered as a group in itself. So, there are totally 3653 groups (as there are 3653 households). Each group (household) contains the immunization status (corrected) of the male-female pair of children present in the group (household). With such a partitioning, the difference in the immunization status of children within a group (household) can be considered as the result of difference of gender of the children. The overall inequality in immunization status is now decomposed into within-group (within-household) and between-group (between-household) components. The resulting within-group component in this decomposition is nothing but the gender based within-household (or within-household) inequality in immunization status.

The overall inequality in immunization status is decomposed into the above mentioned components using mean log deviation as the inequality measure (for similar decompositions, see [77]–[78]). Mean log deviation (MLD) is additively decomposable and can be decomposed meaningfully into two components; first being the within-group component and second the between-group component. Within-group component is nothing but a weighted average of subgroup inequality values and the between-group component is the between-group contribution to overall inequality, representing the level of inequality obtained by replacing the immunization status of each child with the average immunization status of his/her respective group. MLD is also a path independent measure. If the interest is in obtaining the within-group component, it can be obtained in two ways. First, we replace the individual immunization status of each child with a product of individual immunization status and the ratio of overall mean immunization status (of sample) to mean immunization status of his/her group. This operation will suppress all between-group inequality, leaving only inequality within the groups. If MLD is now applied on this “standardized” distribution, it will give the within-group component directly.

Instead, if the immunization status of each child in every group is replaced with the group-specific mean, then all the within-group inequality will be eliminated, and the resulting “smoothed” distribution will have only the between-group component. The within-group component can now be obtained (indirectly) by subtracting the inequality in the aforementioned “smoothed” distribution from the overall inequality in the actual distribution. If the within-group component obtained from the two processes is same, then the inequality measure is considered to be path independent. In addition, MLD also satisfies the four basic properties (anonymity or symmetry; population replication or replication invariance; mean independence or scale invariance; and Pigou-Dalton principle of transfers) applied to inequality measures. It is worth noting that MLD is the only inequality measure which satisfies the above six properties (four basic properties and the properties of subgroup additive decomposability and path independence). The literature on inequality measures and the properties of the inequality measures are fairly developed and the details can be obtained from the past studies [79]–[84]. The form of MLD and the mathematical details of the decomposition procedure are provided in **Appendix S1**.

## Results

The mean immunization status was 2.10 and 2.70 in 1992–93 and 2005–06 respectively (Table 1). The immunization status of boys was better than girls in both the years. The regional variations in average immunization status of children were marked with children from southern and western regions having better status than the other regions. Findings further reveal that boys had better immunization status than girls in all the specified geographic regions of India. Of note is the finding that the differences in the average immunization status for boys and girls were much starker in 1992–93 compared to 2005–06.

**Table 1:** Mean immunization status in the sample by gender and regions, 1992–2006^1^.

Regions	1992–93			2005–06		
	Boys	Girls	All	Boys	Girls	All
North	2.67	2.40	2.54	2.98	2.90	2.94
	(468)	(468)	(936)	(698)	(698)	(1396)
Central	1.76	1.44	1.60	2.54	2.51	2.52
	(471)	(471)	(942)	(843)	(843)	(1686)
East	1.73	1.40	1.56	2.58	2.48	2.53
	(256)	(256)	(512)	(529)	(529)	(1058)
North East	1.17	1.11	1.14	2.14	2.13	2.14
	(226)	(226)	(452)	(675)	(675)	(1350)
West	2.87	2.80	2.84	3.21	3.11	3.16
	(232)	(232)	(464)	(393)	(393)	(786)
South	2.92	2.83	2.87	3.29	3.19	3.24
	(281)	(281)	(562)	(515)	(515)	(1030)
India	2.21	1.99	2.10	2.73	2.67	2.70
	(1934)	(1934)	(3868)	(3653)	(3653)	(7306)

1 Sample size in parenthesis.

### Household fixed-effects analysis

The coefficients estimates from the ordinary least square analysis (with household fixed-effects) are shown in Table 2. At the all India level, the immunization status of girls was significantly lower than the boys in 1992–93 as well as in 2005–06. However, the negative effect of being a “female” was much larger in 1992–93 compared to that in 2005–06.

**Table 2:** Ordinary least square estimates (95% confidence intervals) of multiple liner regression models of the dependent variable “Immunization status” with household fixed effects.

	1992–93	2005–06
Female	−0.21 (−0.27, −0.15)	−0.07 (−0.10, −0.04)
Birth order^1^	0.02 (−0.14, 0.19)	−0.07 (−0.16, 0.02)
Age (in months)^2^	0.03 (0.01, 0.05)	0.01 (0.00, 0.02)
Square of Age	0.00 (0.00, 0.00)	0.00 (0.00, 0.00)
Constant	1.99 (1.19, 2.79)	2.79 (2.39, 3.18)
N	3868	7306

1 Mean Birth order (1992–93) = 3.03; mean birth order (2005–06) = 2.78.

2 Mean age (1992–93) = 29.16 months; mean age (2005–06) = 35.58 months.

### Gender based within-household inequality in immunization status

The total inequality in immunization status of children in India reduced from 0.225 in 1992–93 to 0.140 in 2005–06 (Table 3). A similar trend is observed for all the six regions.

**Table 3:** Gender based within-household inequality in immunization status: All India and regions (1992–93 and 2005–06)^1^.

	1992–93^a^				2005–06^b^			
Inequality →Regions ↓	Total^2^(1)	WH^3^(2)	BH^4^(3)	WH/Total(%)(4)	Total(5)	WH(6)	BH(7)	WH/Total(%)(8)
North	0.1949	*0.0309*	0.1640	*15.86*	0.1335	*0.0213*	0.1122	*15.98*
Central	0.2491	*0.0462*	0.2030	*18.54*	0.1081	*0.0150*	0.0931	*13.91*
East	0.2383	*0.0492*	0.1891	*20.66*	0.1484	*0.0295*	0.1188	*19.89*
North East	0.2602	*0.0334*	0.2268	*12.83*	0.2299	*0.0385*	0.1913	*16.76*
West	0.1376	*0.0189*	0.1187	*13.71*	0.0896	*0.0149*	0.0748	*16.59*
South	0.1432	*0.0238*	0.1194	*16.64*	0.0852	*0.0185*	0.0666	*21.78*
India	0.2250	*0.0349*	0.1901	*15.50*	0.1404	*0.0232*	0.1172	*16.49*

1 Based on Mean Log Deviation estimates.

2 Total stands for total inequality.

3 WH stands for within (intra) – household inequality. It is nothing but the absolute level of gender based within-household inequality (GWHI).

4 BH stands for between (inter) – household inequality.

a Inequality has been estimated on Immunization status corrected for age and birth order of children. That is, the residuals from the following regression (1992–93):
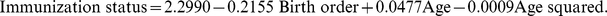
Since the residuals are centered around zero, they have been added a constant (3.0933) in order to match the actual series. The corrected immunization scores are always greater than zero.

b Inequality has been estimated on Immunization status corrected for age and birth order of children. That is, the residuals from the following regression (2005–06):
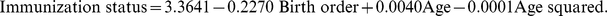
Since the residuals are centered around zero, they have been added a constant (3.3806) in order to match the actual series. The corrected immunization scores are always greater than zero.

The estimates of gender based within-household inequality (GWHI) in immunization status are reported in both, the absolute terms (columns 2 and 6) and as a percentage of total inequality (columns 4 and 8). The absolute level of GWHI at the all India level also decreased from 0.035 in 1992–93 to 0.023 in 2005–06. Barring the northeastern region, all other regions showed a decrease in the absolute level of GWHI during 1992–93 to 2005–06. In 2005–06, western region showed the lowest GWHI in absolute terms whereas northeastern region had the highest.

However, GWHI as a percentage of total inequality increased marginally at the all India level. The figures for 1992–93 and 2005–06 stood at 15.5 and 16.5%, respectively. It may also be noted that, GWHI as a percentage of total inequality decreased in the central and eastern regions but increased in the northeastern, western and southern regions. In the northern region, it remained at the same level. In 1992–93 the GWHI as a percentage of total inequality was highest in the eastern region; it was also at the higher side in 2005–06. But, in 2005–06, the GWHI as a percentage of total inequality was highest in the southern region. It is worth noting that, GWHI as a percentage of total inequality being highest in the southern region should be seen in the light of the fact the total inequality itself was lowest in the southern region. GWHI as a percentage of total inequality was found to be the lowest in the central region in 2005–06. It is not at all a surprising finding given the fact that the average immunization status was quite low for both boys and girls in this region. Surprisingly, as a percentage of total inequality, the northern region had the second lowest level of GWHI in 2005–06.

## Discussion

The present study for the first time presents time-trends in GWHI in providence for childhood immunizations in India and its six specified geographical regions. It also for the first time, using novel statistical and decomposition techniques, brings to the forefront the extent of GWHI in immunization status of Indian children and supports the earlier debate on with-in household discrimination against the female children. The findings clearly suggest substantial GWHI in immunization status of children, even in 2005–06. Though the overall inequality in immunization status of children had declined in all the specified geographic regions, the changes in GWHI were mixed.

This study found that the gender based inequality in immunization within households as a percentage of total inequality in immunization has increased by one percentage point at the all India level during the period 1992–93 to 2005–06. It has happened even though, in absolute terms, both the overall inequality and the GWHI have decreased. The decrease in the overall inequality and the absolute level of GWHI were 37.6 and 33.5%, respectively. The mean immunization status of Indian children also increased during the aforementioned period. This points towards two things; first the various programmes implemented by the government of India to increase the awareness about the need for immunization and its providence have shown results. But the gender discrimination in providence for immunizations has not decreased at the same rate as other factors. This is so because the GWHI has decreased at a rate slower than the decrease in overall inequality.

As the present study has used household fixed effects and household based inequality decomposition analyses which is a departure from the existing studies, it is important to briefly discuss the advantages which these analyses offer over the more conventional analyses used in the earlier studies. In multiple regression analyses where the primary focus is to identify the kind of relationship between an outcome variable and child gender, the estimates may be biased if the household fixed effects are not used. This may happen because; even though the analyses include a number of household level controls (for example, parental education and household wealth) there is always a possibility of the existence of some unobservable household level characteristics correlated with child gender which are not included in the analyses. In such situations, the coefficient of the variable “child gender” is likely to be biased. Whereas, the use of household fixed effects makes it possible to control for all the observed and unobserved household-level variables. This eliminates the possibility of bias in the estimates due to omission of some observed or unobserved household level variables.

Similarly, the decomposition of the overall inequality in an outcome measure for the children of the two sexes (after controlling for age and birth order), into within-household and between-households components also offers additional advantages. Earlier studies (for example, [8], [15], [18], [62]) on gender based differentials in health care (including immunization) for children, have used logistic regression models. These studies reported that there is gender based discrimination in health care for children because the odds of male children receiving health care was higher than that of the female children. This kind of analysis takes into account the comparison of all female children with all the male children in the sample, that is, it compares a female child of one household not only with the male children in the same household but also with the male children of other households and vice versa. Though the logistic regression models used by the above mentioned studies include multiple controls (for example, parental education, household wealth, caste, religion etc.) which vary across households, there can always be unobserved determinants varying across households which affect the measured health care variable (outcome measure) for the children. In this case there is always a possibility that the odds ratio for the variable “gender” in addition to capturing the “gender” effect also captures the effects of the unobserved determinants varying across the households. Therefore, using this kind of analysis (odds ratio for the variable “gender”), it is difficult to infer about the extent of direct discrimination between girls and boys within the households.

Whereas, inequality decomposition based analysis presented in this paper directly informs about the extent of disparity in the immunizations received by children due to discrimination between boys and girls within the households. The within-household component of the total inequality in immunizations received by children only captures the inequality in immunizations received by children within the households (weighted sum of the inequalities in individual households). Since, the household level characteristics which affect the immunizations received by children are common for both the children in a household and the child level characteristics (birth order and age) except gender which vary across children in a household and which affect the immunization status of children are controlled for, the inequality between the immunization status of the female child and the male child (within the household) can be safely attributed to the difference in their sexes. Also, as the overall inequality among children in the sample is an exact sum of the within household and the between household components, one can safely estimate the proportion of total inequality among children which is due to gender related discrimination inside the households.

Though the present study has several advantages it also suffers from a few limitations. The first one being that, it is silent on the statistical significance of the changes in the GWHI in the immunization status of children over time. This is not a major limitation because this measure is similar to other common poverty and inequality indices measuring the welfare of a population, for example head count ratio (for measuring poverty), which remain silent on the statistical significance of the changes over time. Using them, one can at best comment on the extent of (percentage) increase or decrease in the measured outcome over time. The second limitation can be thought of in the sense that the eligible sample is a subsample of the overall sample of children, but this also is likely to introduce a very small bias in the analysis presented because the sex ratio in the sample of excluded children is not very different from the sex ratio in the sample used for the analysis.

The findings of the study are of potential value and are indicative. For example, scholars have argued that with declining fertility levels and with the advancement of sex-detection technologies, one would expect that the post-natal discrimination against the female children gets converted into prenatal discrimination and the female children thus born should get equal attention and the discrimination against female children should go down [84]. However, the findings of this study do not suggest any decline in GWHI as a percentage of total inequality except for the central region (a less than one percentage point decrease was also observed in the case of eastern region). On the other hand, northern, northeastern, southern and western regions noted an increase. It may be noted that the increase in case of southern and western region could be simply due to the higher decrease in overall levels of inequality than the decrease in within-household component. It is disheartening to note that even these otherwise economically and socially advanced geographic regions are not free from gender discrimination when it comes to providence for preventive health care.

Last but not the least, United Nations Millennium Development Goal (MDG) four “Reduce Child Mortality” aims to reduce under-five mortality by two thirds by 2015 [85]. As the vaccine-preventable diseases are responsible for nearly 20% of the 8.8 million deaths occurring annually among children under five years of age, immunization can significantly contribute to achieving this goal [63]. Further, immunization is one of the most successful and cost-effective public health investments. In addition, immunization leads to significant economic benefits as it protects individuals not only against getting an illness but also against the long-term effects of that illness on their physical, emotional and cognitive development. When children grow up healthier, they do better in school and are more productive as adults [63]. Therefore, it is critical that government of India places investing in immunization high on their national health agenda. Since in India boys are preferred over girls when it comes to provision for health care which includes immunization, the achievement of the above mentioned MDG by India will depend on whether the Government of India is able to create an atmosphere where parents pay equal attention to immunization of both, boys as well as girls. As, the studies on the Indian subcontinent [86]–[88] have shown that the effectiveness of immunization programmes can be increased through strengthening of health systems, better planning and management, enhancing political commitment, and mass campaigns raising the awareness among the masses; it is high time, Government of India integrates the child immunization initiatives to the various health care programmes and campaigns on health related issues in India.

## Supporting Information

Appendix S1 MLD and the decomposition process.
(DOC)
