# Correction: Gender Based Within-Household Inequality in Childhood Immunization in India: Changes over Time and across Regions

**DOI:** 10.1371/journal.pone.0173544

**Published:** 2017-03-03

**Authors:** Ashish Singh

The equation in the file S1 Appendix is referenced incorrectly. Please see the correct S1 Appendix below.

## Supporting information

S1 Appendix MLD and the decomposition process. (DOC)

## References

[1] Singh A (2012) Gender Based Within-Household Inequality in Childhood Immunization in India: Changes over Time and across Regions. PLoS ONE 7(4): e35045 doi: 10.1371/journal.pone.0035045 22509379 10.1371/journal.pone.0035045 PMC3324412

