# Supplementary material for: Correction: Gender Based Within-Household Inequality in Childhood Immunization in India: Changes over Time and across Regions
Source: PLoS One. 2017 Mar 3;12(3):e0173544. doi: 10.1371/journal.pone.0173544 (PMC5336290; doi:10.1371/journal.pone.0173544)
Supplement: S1 Appendix — (DOC) [file pone.0173544.s001.doc]

**Appendix S1.** MLD and the decomposition process

The decomposition procedure used for decomposing the overall inequality in immunization status into within-households and between-households (within-groups and between groups; each household is taken as a group) components is as follows:

Let the mean log deviation (MLD) be represented by *M*, and suppose that the children, *N*, are partitioned into *m* proper subgroups *N_k_* (*k = 1,2, …,m*) based on their household, with respective immunization status vectors , mean immunization status , population sizes , and population shares . Also, let , denote the distribution obtained by replacing each immunization status in the vector with the subgroup mean, . Then (following [82]),

= *W* + *B*

where, *W* is the within-group (within-household) inequality and *B* represents the between-group component. *W* is nothing but a weighted average of subgroup inequality values and *B* is the between-group contribution to inequality, representing the level of inequality obtained by replacing the immunization status of each child with the mean immunization status of their respective subgroup (household).
